# The Feasibility and Safety of Endoscopic Submucosal Dissection for Circumferential Superficial Esophageal Squamous Cell Neoplasms

**DOI:** 10.3390/jcm12020471

**Published:** 2023-01-06

**Authors:** Yi Liu, Lizhou Dou, Wei Rao, Yong Liu, Yueming Zhang, Shun He, Liyan Xue, Guiqi Wang

**Affiliations:** 1 Department of Endoscopy, National Cancer Center/National Clinical Research Center for Cancer/Cancer Hospital, Chinese Academy of Medical Sciences and Peking Union Medical College, Beijing 100021, China; 2 Department of Pathology, National Cancer Center/National Clinical Research Center for Cancer/Cancer Hospital, Chinese Academy of Medical Sciences and Peking Union Medical College, Beijing 100021, China

**Keywords:** circumferential lesions, superficial esophageal squamous cell neoplasm (SESCN), endoscopic submucosal dissection (ESD), surgery, deep submucosal invasion, angiolymphatic invasion

## Abstract

Background It remains controversial whether endoscopic submucosal dissection (ESD) is still appropriate for circumferential superficial esophageal squamous cell neoplasms (SESCN), and few studies compared the short-term and long-term outcomes of ESD with radical surgery. Methods A total of 140 patients with SESCN who underwent ESD or surgery between February 2014 and October 2021 were retrospectively reviewed. The characteristics of patients, operative time, postoperative complications, overall survival (OS), recurrence-free survival (RFS), and quality of life (QOL) were compared between the ESD and surgery groups. The effect of different methods to prevent esophageal stenosis after ESD were analysed. Results Drinking, family history of cancer, macroscopic type, and intrapapillary capillary loop (IPCL) type were independent risk factors for deep submucosal invasion (SM ≥ 200 μm). Smoking and IPCL type were independent predictive factors for angiolymphatic invasion. The average operative time of ESD was significantly shorter than that of surgery (174.5 ± 51.16 min vs. 255.9 ± 88.18 min, *p* < 0.001). The incidence of perioperative complications in ESD group was significantly lower than that in surgery group (5.5% vs. 19.4%, *p* = 0.015). The ESD group had significantly better functional scale scores for emotional functioning, cognitive functioning, and global health status, and lower rates of pain, dyspnoea, insomnia, appetite loss, diarrhoea, reflux, and trouble with taste than the surgery group. No significant difference in OS and RFS between ESD and surgery group. Conclusions ESD can significantly shorten the operative time and reduce perioperative complications. Additionally, on the premise of using appropriate measures to prevent postoperative stenosis, ESD can be the first choice for the treatment of SESCN, which could provide better QOL, and the long-term prognosis of ESD is no less than that of surgery.

## 1. Introduction

Esophageal squamous cell carcinoma has become the sixth most commonly occurring cancer in China, and its mortality rate ranks fourth among all kinds of cancers in China [1]. In recent years, with the development of endoscopic diagnostics, the number of superficial esophageal squamous cell neoplasms (SESCNs, including squamous cell carcinoma and precancerous lesions) detected at an early stage has increased. Currently, the treatment of SESCN mainly includes endoscopic resection (ER) and surgical resection, but both have their own limitations. Although surgical resection is an effective and radical treatment for SESCN, the incidence of postoperative complications such as bleeding, anastomotic fistula, pulmonary infection, chylothorax, empyema, functional gastric emptying disorder, reflux esophagitis, and anastomotic stricture is high. In addition, surgical resection causes major surgical trauma, and patients typically experience a poor quality of life postoperatively. Relevant studies have reported that the 5-year survival rate of patients with SESCN located within the mucosa and/or submucosa undergoing ER is approximately 85–95%, which is comparable to that of surgery [2,3]. Moreover, ER is associated with fast recovery, minimal trauma and better postoperative quality of life (QOL) for patients due to esophagus preservation.

With the continuous development and improvement of endoscopic mucosal dissection (ESD), the size of the SESCN is no longer a limitation for ER. Thus, near-circumferential lesions, or even entire circumferential lesions, can be removed en bloc by ESD. However, if the SESCN involves the circumference of the lumen, then the problem of postoperative esophageal stricture after ESD cannot be ignored. Therefore, the indications for ER are still controversial. According to the 2020 Japanese endoscopic submucosal dissection/endoscopic mucosal resection guidelines for esophageal cancer, ER is less recommended for cT1a-EP/LPM superficial squamous cell carcinomas with a major axis length of 50 mm and involving the entire circumference of the esophagus upon implementing preventive measures for stenosis [4]. Although there are several strategies for prevention and treatment in post-ESD esophageal stricture [5,6,7,8,9], the occurrence of other postoperative complications, including perforation, massive haemorrhage, and postoperative infection, is also closely related to the size of the lesion, the depth of invasion, and the extent of resection of the lumen [10,11].

Due to rich submucosal lymph vessels, the deeper the depth of invasion, the higher the risk of angiolymphatic invasion and lymph node metastasis [12,13]. As a result, ER is generally indicated for patients with very low or no risk of lymph node metastasis. It is particularly important to evaluate the invasion depth of the SESCN and the status of angiolymphatic invasion during preoperative endoscopy. Few reports have analysed the clinicopathological characteristics of circumferential SESCN, indicated how to screen for lesions with a low risk of deep submucosal invasion or angiolymphatic invasion or determined whether they meet the criteria for the ESD procedure. Therefore, the aim of our study was to investigate the risk factors associated with the depth of tumour invasion and angiolymphatic invasion. Meanwhile, we compared the short-term and long-term outcomes of ESD versus surgery for SESCN and evaluated the benefits of ESD on the improvement of QOL for those patients, in order to assess the feasibility and safety of the ESD procedure for circumferential SESCN.

## 2. Methods

### 2.1. Patients

**Figure 1:**
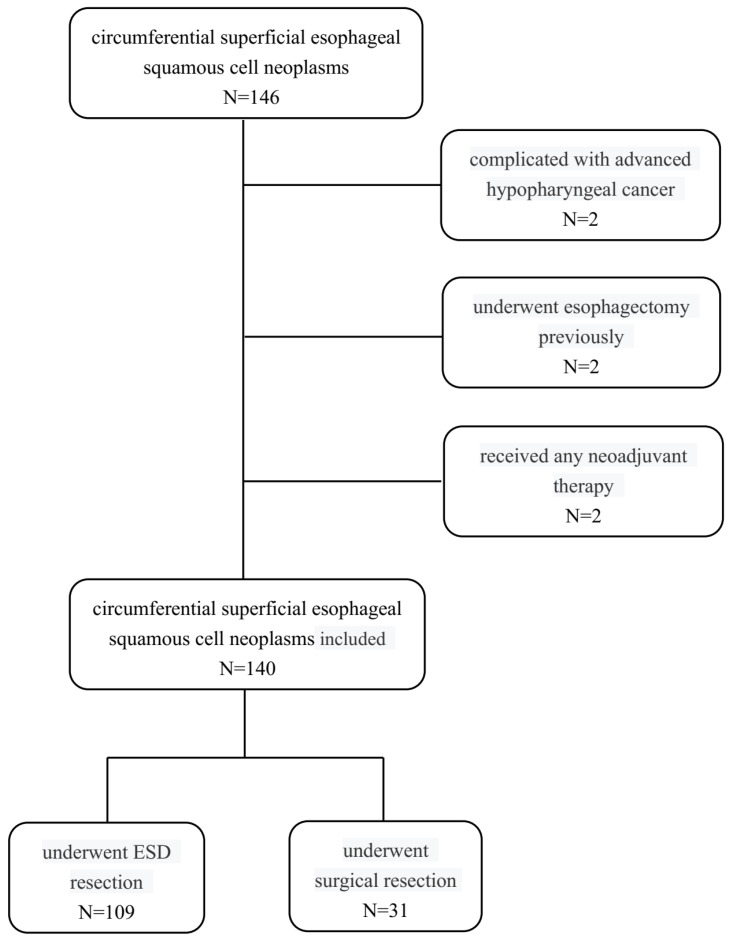
Flow chart of the current study.

This study included 146 consecutive patients with SESCN who underwent ER and surgical resection at Cancer Hospital, Chinese Academy of Medical Sciences and Peking Union Medical College between February 2014 and October 2021. The inclusion criteria were as follows: (1) high-grade intraepithelial neoplasia or squamous cell carcinoma of circumferential lesions confirmed by pathology; (2) preoperative chest or/and abdomen CT showing no definite thoracic lymphadenopathy or metastasis; and (3) complete preoperative and postoperative clinical and pathological data. Patients were excluded if they (1) had other advanced malignancies in other sites; (2) underwent esophagectomy previously; (3) received any neoadjuvant therapy; or (4) had a serious cardiovascular or cerebrovascular disease, liver and kidney dysfunction, severe blood system diseases, immune system diseases, or severe mental disorders. Finally, a total of 140 patients met the above criteria and were selected as participants (Figure 1). The patients were divided into an ESD group and a surgery group according to patient’s preference after full communication with the patient. The study was conducted in accordance with the Declaration of Helsinki (as revised in 2013). This study was approved by the Ethics Committee of the National Cancer Center/Cancer Hospital, Chinese Medical College and Peking Union Medical College (No. 19/191-1975), and written informed consent was obtained from all the patients before the operation.

### 2.2. Evaluation Parameter

All patients underwent white light endoscopy (WLE) and magnifying endoscopy with narrow band imagining (ME-NBI) to estimate their lesions. EUS was performed to estimate the depth of lesions, if the patient was tolerant to it. Then, iodine staining was performed using 1.25% Lugol’s solution to further determine the extent of the lesion. The baseline characteristics and short- and long-term outcomes of patients were compared between the ESD and surgery groups. The baseline characteristics of patients included age, sex, body mass index (BMI), family history of tumour, smoking history, drinking history, lesion location, lesion size, macroscopic type, intrapapillary capillary loop (IPCL), histological differentiation degree, depth of invasion, and comorbidities. The lesion location was classified according to the 8th edition of the esophageal TNM staging criteria of Union for International Cancer Control/American Joint Committee on Cancer (UICC/AJCC). The upper part is less than 25 cm away from the incisors, and the middle section is 25–30 cm away from the incisors, while the lower section is ≥30 cm away from the incisors. The macroscopic types and depth of invasion were classified according to the Paris endoscopic classification of superficial neoplastic lesions [14]. IPCL classification was based on the classification standard of Haruhiro Inoue and Japan esophageal society (JES) [15,16]. The short-term outcome measures were the rates of en bloc and complete resection in the ESD group, operative time, and perioperative complications. En bloc resection was defined as resection of a lesion in one piece. Complete resection was defined as a resected specimen with tumour-free lateral and vertical margins. The operative time was defined as the total time of ESD or surgery procedures. Perioperative complications included bleeding, perforation, anastomotic fistula, esophageal scar stenosis, and anastomotic stricture. Long-term outcomes included overall survival (OS), recurrence-free survival (RFS), and quality of life after the ESD or surgery procedures. OS was defined as the period before death. RFS was defined as the period before any type of recurrence. QOL was assessed by the validated Chinese version of the European Organization for Research and Treatment of Cancer (EORTC) Quality of Life Questionnaire-Core 30 (QLQ-C30) and Quality of Life Questionnaire-OES18 (QLQ-OES18) 6 months after the treatment or at the end of follow-up.

### 2.3. Operation Procedure

ESD group: The Japanese Olympus GIF-Q260J electronic gastroscope was used for ESD treatment. The specific steps are as follows: (1) Endoscopic marking: after esophageal lesions were stained with 1.25% Lugol’s solution, a dual knife (KD-650Q, Olympus, Japan) was used to mark 5 mm outside the lesion; (2) Tunnel establishment: the tunnel entrances were established at the 12 and 6 o’clock positions on the oral side of the lesion, submucosal injection was performed with an injection needle (NM-200 L-0523, Olympus, Japan), and a preincision was made outside the marked point on the oral side of the lesion; (3) Circumferential incision of the lesion’s anal mucosa to prepare for rendezvous; (4) Gradually perform submucosal dissection along both sides of the tunnel from the side of the lesion until it joins with the anus, leaving only the mucosa at the entrance of the tunnel; (5) The mucosa at the entrance of the tunnel was peeled off, and the specimen was removed; (6) Electrocoagulation and haemostasis were performed on the exposed blood vessels and active bleeding at the wound using electrobiopsy forceps (FD-410LR, Olympus, Japan). 109 patients of present study were categorized into five groups according to different interventions to prevent esophageal stenosis, such as repeated endoscopic balloon dilation (EBD), polyglycolic acid (PGA) felts (Neoveil, 100 × 50 × 0.15 mm; Gunze Co., Tokyo, Japan) with autologous esophageal mucosa (AEM), PGA with temporary stent implantation (TSI), PGA with AEM transplantation and TSI, and PGA with AEM transplantation and self-control stricture-preventing water balloon (SSWB). 50 patients received repeated EBD and 2 PGA felts with AEM were positioned on the surface of the ulcer. Meanwhile, 48 patients received TSI. We measured the length of the ulcer endoscopically after resection, and stents with appropriate lengths were selected. Then, 43 PGA felts with AEM tissues and 5 PGA felts without AEM tissues were made onto a covered metal mesh stent (CMMS) (MTNSE-S-20/160-A-8/650, MTN-SE–S-20/100-A-8/650, MTN-SE–S-18/120-A-8/650; Nanjing Micro Technology Co., Ltd., Nanjing, China). The endoscope was passed through the stent with grasping forceps through the biopsy channel to grasp the distal steel lasso loop of the stent. Eventually, the stent was positioned on the surface of the ulcer. Before stenting, an overtube was placed through the mouth (MD-48618, Sumitomo Bakelite Co., Ltd., Tokyo, Japan) to facilitate stent passage and protect the laryngopharyngeal mucosa from injury. Besides, 9 PGA felts with AEM tissues were made onto SSWB, and finally they were positioned on the surface of the ulcer.

Surgery group: Radical esophagectomy and two (mediastinal and perigastric) or three (cervical, mediastinal, and perigastric) fields of regional lymphadenectomy were routinely performed. The anastomotic site was related to tumour location. In general, cervical anastomosis was performed in patients with upper esophageal tumours. A supra-aortic arch esophagogastric anastomosis was performed for patients with middle or lower esophageal lesions. Reconstruction of the alimentary tract was performed using the stomach or jejunum.

### 2.4. Postoperative Management and Follow-Up

ESD group: Patients with esophageal stent implantation were asked to continue fasting for the first three days after ESD and then underwent CT on the 3rd day to determine whether there were perforations or stent migrations. On the 4th day after ESD, the patients were allowed to start a liquid diet, and on the 7th day, the gastric tube was removed. After ESD, the patient received a proton pump inhibitor (rabeprazole 20 mg; Changao, Nanjing, China) for 6 consecutive days and antibiotics for 3 days q12 h (amoxicillin sodium sulbactam sodium 1.25 g; Ruiyang, Shandong, China). A scheduled endoscope examination was performed once a week to confirm the position of the stent, which was removed during the 6th–8th week after ESD depending on the patient’s tolerance. Patients were examined by endoscopy 2 weeks and 3 and 6 months after stent removal and then annually. Patients without esophageal stent implantation were asked to continue fasting for the first three days after ESD and received routine nutritional support, protection of gastric mucosa, inhibition of gastric acid secretion and indwelling gastric tubes for gastrointestinal decompression. The mice were fed liquid food for 3 days and gradually transitioned to a normal diet. Patients returned to the hospital 1 month after ESD for re-evaluation by endoscopy, and the gastric tube was removed. Patients were examined by endoscopy 1, 2, 3, and 6 months after ESD and then annually. When the standard endoscope (GIF-H290; Olympus, Tokyo, Japan) cannot pass through the stenosis, it is defined as esophageal stricture (ES). Patients with ES underwent regular endoscopic esophageal balloon dilation until the standard endoscope (GIF-H290; Olympus, Tokyo, Japan) could pass through the esophageal stenosis during re-examination.

Surgery group: Routine fasting was performed after the operation. A nasogastric tube was left postoperatively for gastric decompression, and an indwelling gastrointestinal tube was left postoperatively for enteral nutrition. The gastric tube was removed 3 days after the operation, and liquid food was started after 2 weeks of nasogastric feeding and gradually transitioned to a normal diet. Patients were re-examined by endoscopy 3 and 6 months after surgery and then annually thereafter.

### 2.5. Statistical Analysis

The statistical calculations were conducted employing SPSS 22 software (SPSS Inc., Chicago, IL, USA). Independent-samples *t*-test was used to compare continuous variables that were normally distributed. Nonparametric *Mann–Whitney U* tests were used when variance was not normally distributed. The chi-square test or *Fisher’s* exact probability method was used for the comparison of classified variables between two groups. Independent risk factors were analysed by univariate and multivariate logistic regression. The *Kaplan–Meier* method was used for survival analysis. A *log-rank* test was used for comparison of survival curves. *p* < 0.05 was considered to be significantly different.

## 3. Results

### 3.1. Patients and Clinicopathological Features

A total of 140 patients (95 males, 45 females; mean age 62.74 years, range 45–85) were enrolled, and 109 patients were treated with ESD, while 31 patients were treated with surgery. Patient and tumour characteristics are summarized in Supplemental Table S1. Lesions were detected in the upper esophagus in 11 patients, in the upper-middle esophagus in 24 patients, in the middle esophagus in 41 patients, in the middle-lower esophagus in 54 patients, and in the lower esophagus in 10 patients. The overall median longitudinal diameter of the lesions was 70 mm (30–190 mm). Regarding the macroscopic types, 43 lesions were type 0-IIa, 96 were type 0-IIb, and 1 was type 0-IIc according to the Paris endoscopic classification [17]. Of the 140 lesions, 118 (84.3%) were carcinomas and 22 (15.7%) were high-grade intraepithelial neoplasias (HGINs).

### 3.2. Preoperative Endoscopic Ultrasonography (EUS) Findings

Most patients (130 of 140 patients) underwent preoperative EUS (Supplemental Table S2). Only 77 (59.2%) were consistent with the depth of postoperative pathological diagnosis. Preoperative EUS accounted for 19.7% of patients with underdiagnosis of invasion depth and up to 59.4% with overdiagnosis (Supplement Table S2). For lesions infiltrating the submucosa (≥SM) assessed by preoperative EUS, the risk of postoperative pathological infiltration into the submucosa was 2.789 times that of the preoperative assessment of lesions confined to the mucosal layer (<SM) (Supplement Table S3). 

### 3.3. The Relationship between Clinicopathological Data and Depth of Invasion/Angiolymphatic Invasion

In the univariate regression models, smoking history, drinking history, family history of cancer, lesion location, macroscopic type, slightly elevated/depressed (WLE), and IPCL (JES classification) were significantly related to deep submucosal invasion (SM ≥ 200 μm), while smoking history, drinking history, complications with early laryngeal tumour, macroscopic type, slightly elevated/depressed (WLE), IPCL (JES classification), and deep submucosal invasion (SM ≥ 200 μm) were significantly related to angiolymphatic invasion (*p* < 0.05). (Table 1 and Table 2).

Multivariate stepwise logistic regression analysis revealed that drinking history, family history of cancer, slightly elevated/depressed (WLE) and IPCL (JES classification) were independent predictive factors for deep submucosal invasion (SM ≥ 200 μm). Meanwhile, smoking history and IPCL (JES classification) were independent predictive factors for angiolymphatic invasion (Table 1 and Table 2).

### 3.4. Comparison of Clinicopathological Characteristics between the ESD and Surgery Groups

There were no statistically significant differences in age, sex, BMI, lesion location, longitudinal diameter, macroscopic type, pathological type, angiolymphatic invasion, or nerve invasion between the ESD group and the surgery group (*p* > 0.05). The difference between the ESD group and the surgery group was statistically significant in the depth of invasion (*p* < 0.05). The lesions in the ESD group were mainly from EP to SM (<200 μm), while the deep submucosal invasion (SM ≥ 200 μm) rate of the lesions in the surgery group was higher (Table 3). 

### 3.5. The Short-Term Outcomes and Long-Term Outcomes of ESD and Surgery

The main treatment outcomes are summarized in Table 4. All patients achieved en bloc resection. The complete resection rate was 99.1%. The average operative time of ESD (174.5 ± 51.16) was significantly shorter than that of surgery (255.9 ± 88.18) (*p* < 0.001). The rate of perioperative complication was significantly higher in the surgery group than in the ESD group (19.4% vs. 5.5%, *p* = 0.015). Immediate perforation occurred in one patient during ESD, and it was successfully closed after the application of titanium clips without other severe complications. Delayed perforation occurred in two patients in the ESD group, and both patients recovered after conservative medical treatment. There were three patients in the ESD group who developed delayed bleeding postoperatively, all of whom underwent endoscopic haemostasis, and no severe complications regarding bleeding were observed. Anastomotic fistula occurred in one patient in the surgery group, and it was cured after conservative treatment. There were five patients in the surgery group suffering from wound infection, which healed after multiple dressing changes. Esophageal scar stenosis occurred in 85 patients after ESD. Anastomotic stricture occurred in four patients after surgery.

**Figure 2:**
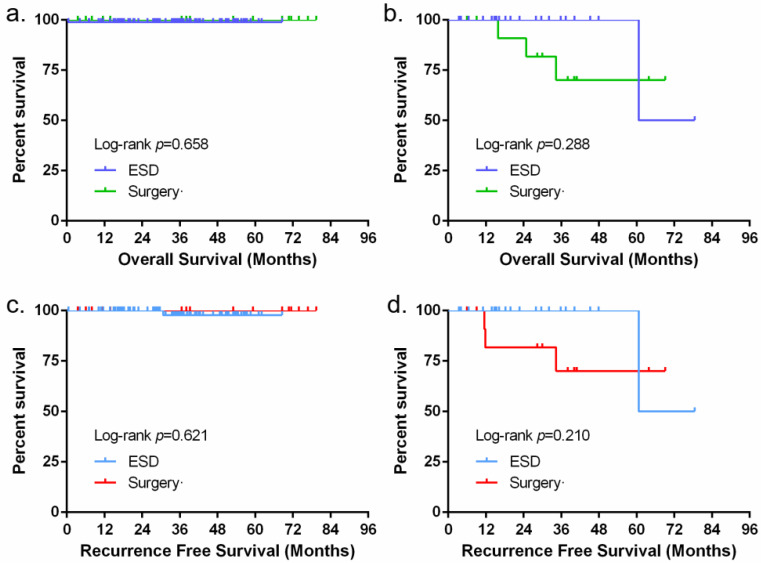
(**a**) Kaplan–Meier curve of overall survival for depth of invasion (<SM1); (**b**) Kaplan-Meier curve of overall survival for depth of invasion (≥SM1); (**c**) Kaplan–Meier curve for recurrence-free survival for depth of invasion (<SM1); (**d**) Kaplan-Meier curve for recurrence-free survival for depth of invasion (≥SM1).

The median follow-up times were 29.7 months (range 3.38–78.52) and 39.3 months (range 3.75–79.51) in the ESD and surgery groups, respectively. In the ESD group, 29 patients were pathologically reported to have deep submucosal invasion (SM ≥ 200 μm) or angiolymphatic invasion. Of them, eight patients received radiotherapy, three patients received surgery, and the remaining patients did not undergo any additional treatment. During follow-up, one patient presented with a local recurrence 2 years after ESD and was cured by secondary endoscopic therapy. Another patient with deep submucosal invasion (SM ≥ 200 μm) and angiolymphatic invasion pathologically after ESD who refused any additional treatment died of distant metastasis 5 years postoperatively. Perioperative nonoperative-related death was found in one patient who underwent ESD. In the surgery group, three patients died due to tumour recurrence 1–3 years postoperatively (Table 4). The overall survival time and recurrence-free survival time of both groups were not significantly different (Figure 2).

### 3.6. Comparison of the QOL Scores between ESD and Surgery

In terms of the mean EORTC-QLQ-C30 functional scores, the ESD group had significantly better functional scales for emotional functioning, cognitive functioning and global health status than the surgery group. According to the symptoms scales of the EORTC-QLQ-C30 and the EORTC-QLQ-OES18, patients in the surgery group had significantly higher rates of pain, dyspnoea, insomnia, appetite loss, diarrhoea, reflux, and trouble with taste than those in the ESD group (Table 5).

### 3.7. The Effect of Different Methods to Prevent Esophageal Stenosis after ESD

**Figure 3:**
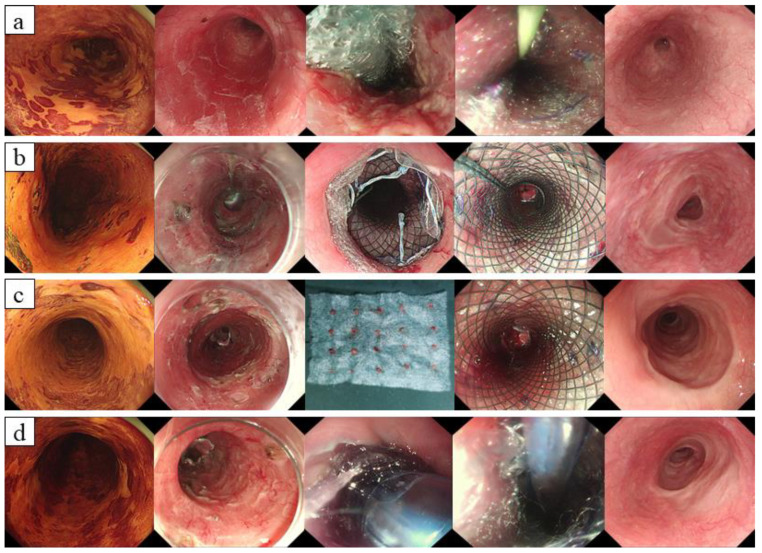
Cases of different methods to prevent esophageal stenosis after treatment. (**a**) PGA with AEM; (**b**) PGA with TSI; (**c**) PGA with AEM transplantation and TSI; (**d**) PGA with AEM transplantation and SSWB.

We categorized patients into five groups according to different interventions to prevent esophageal stenosis, such as repeated EBD, PGA with AEM, PGA with TSI, PGA with AEM transplantation and TSI, and PGA with AEM transplantation and SSWB (Figure 3). PGA with AEM and TSI reduced the mean number of balloon dilatations from 10.8 ± 8.28 (only repeated EBD) to 2.9 ± 4.05 (*p* < 0.001), and the mean number of balloon dilatations was also significantly decreased to 3.1 ± 3.52 in the PGA with AEM transplantation and SSWB group (*p* = 0.008) (Table 6).

## 4. Discussion

We tried to identify clinical and endoscopic characteristics that could help determine which patients with SESCN were good candidates for ER. In this study, we retrospectively reviewed 140 patients with confirmed SESCN who were treated with ER or esophagectomy in our hospital, comprehensively presented their clinical and pathological features and presented several original findings. The results of the multivariate analysis suggested that drinking history, family history of cancer, slightly elevated/depressed (WLE), and IPCL (JES classification) were independent predictive factors for deep submucosal invasion (SM ≥ 200 μm), while smoking history and IPCL (JES classification) were independent predictive factors for angiolymphatic invasion.

Previous studies have reported that the lymph node metastasis rate was almost 0 when SESCN was restricted to the mucosa, but the rate of lymph node metastasis increased to 5.9–14.8% in patients whose tumours invaded the submucosa without vascular invasion and to 25.5–33.3% in patients whose tumours invaded the submucosa with vascular invasion [17,18,19,20,21]. EUS was once considered the most useful modality for judging the depth of lesion invasion. However, previous studies suggested that the accuracy rate of preoperative EUS in judging the depth of invasion was affected by many factors, such as the diameter of lesions greater than 3 cm and the endoscopist’s skill level [22,23]. In addition, approximately 20% of cSM lesions and 30% of cSM1 lesions were pathologically diagnosed as EP or LPM lesions with ER specimens [24]. In this study, it was worth mentioning that up to 59.4% of them had excessive judgement (Supplement Table S1). This may be because the lesions included in our research were all circumferential, and they were often accompanied by inflammation, erosion, etc., which may have affected the endoscopist’s judgement to a certain extent. In addition, some lesions located in the upper esophagus or lower esophagus near the cardia were difficult for the endoscopist to perform EUS. Moreover, a recent systematic review also revealed that the sensitivity and specificity of EUS in determining the depth of invasion of SESCN were similar to those of WLE and ME-NBI, but the overdiagnosis rate of EUS was relatively high [25]. These overdiagnosed patients with circumferential SESCN would be treated with more invasive interventions, such as esophagectomy or definitive chemoradiotherapy, if we did not have our proposed treatment strategy of ER. Therefore, we believe that for SESCN, there are still some limitations in judging the depth of invasion using EUS alone.

As we know, SESCN are often associated with changes in IPCL. Relevant investigation showed that magnification endoscopy observation of IPCLs allowed in vivo discrimination between intramucosal and submucosally invasive cancer [26]. Inoue classification is the most commonly used in clinical practice [15,16]. However, due to the slightly vague concept expression between its various types, the experience of different endoscopists may contribute to differences in the accuracy of the final results. In 2017, Tsuneo Oyama et al. concluded that the overall accuracy of type B microvessels in assessing the depth of invasion of the SESCN was 90.5% in the JES classification [15,16,27]. Moreover, the JES classification was relatively simple and easy for endoscopists to grasp. According to the results of this study, we considered that the JES classification is superior to EUS in judging whether the lesion has deep submucosal invasion clinically. In addition, previous studies showed that non-flat lesions were more likely to infiltrate into the deep submucosa than flat lesions according to the morphology under endoscopy [28,29], which was consistent with our findings (Table 1). In addition, we also found that a family history of tumours and alcohol consumption were independent risk factors for deep submucosal invasion.

It is worth noting that 15.71% (22/140) of patients had angiolymphatic invasions. Previous studies have demonstrated that angiolymphatic invasion is closely correlated with lymph node metastasis [30,31,32,33]. A relevant study showed that angiolymphatic invasion may occur even in stage T1a SESCN, with an incidence of approximately 3.12% [34], which was close to our results (4.29%). Further analysis revealed that T1b SESCN complicated with angiolymphatic invasion accounted for 11.43%, which may be because the esophageal submucosa is rich in vascular tissue, providing sufficient blood supply and lymphatic return to the esophageal mucosa. Notably, the present study found that the risk of angiolymphatic invasion was significantly associated with JES classification. In addition, we also found that smoking was an independent risk factor for angiolymphatic invasion. Interestingly, in a previous study, men were more likely to develop angiolymphatic invasion than women [31], which may be explained by the fact that the ratio of smoking was higher in males than in females.

Although the lesions included in this study were all circumferential esophageal lesions, the ESD procedure achieved high en bloc and complete resection rates (100% and 99.1%, respectively), which were even in line with the noncircumferential lesions in previous research [35]. Thus, we supposed that the size of the lesion is no longer a limiting factor for endoscopic treatment of SESCN. More importantly, ESD was significantly less time consuming and less invasive than surgery and improved the quality of life of patients. In the present research, we used the EORTC QLQ-C30 and EORTC-QLQ-OES18 to compare the effects of ESD and surgery on postoperative QOL in patients with circumferential SESCN. We found that patients in the surgery group had significantly higher rates of pain, dyspnoea, insomnia, appetite loss, diarrhoea, reflux, and trouble with taste than those in the ESD group, which may be caused by large surgical trauma and postoperative anatomical changes. Moreover, the results of the QOL scale showed that the ESD group had significantly better functional scales for emotional functioning, cognitive functioning and global health status than the surgery group. Therefore, compared to surgery, ESD provides postoperative QOL for patients.

In general, postoperative stenosis has become a major concern in terms of long-term outcomes after ESD. Previous studies reported that the incidence rate of esophageal stenosis in patients with wholly circumferential lesions reached 100% [36,37]. Currently, EBD is considered to be an effective treatment for post-ESD stenosis. In addition, several management strategies exist for strictures after esophageal ESD [6,7,8,9]. According to different approaches to prevent postoperative stenosis of the esophagus, we categorized patients into five groups according to different interventions to prevent esophageal stenosis, such as repeated EBD, PGA with AEM, PGA with TSI, PGA with AEM transplantation and TSI, and PGA with AEM transplantation and SSWB. It is worth noting that PGA with AEM and TSI could lessen esophageal stenosis occurrence to 53.5%, and PGA with AEM transplantation and SSWB reduced stenosis incidence in 55.6% of patients. Moreover, the mean number of balloon dilatations of these two methods was significantly less than that of repeated EBD alone. Therefore, our experience may offer some alternatives to decrease the risk of esophageal stenosis for ESD in circumferential SESCN. For patients who underwent ESD in this research, all survived well except for one patient who refused any additional treatment postoperatively; the patient died of distant metastases 5 years after ESD. Comparatively, three patients died due to tumour recurrence 1–3 years postoperatively in the surgery group. In addition, the depth of submucosal invasion did not influence overall survival or recurrence-free survival in the ESD and surgery groups.

To our knowledge, this is the first study to compare the QOL and efficacy of ESD versus surgery for circumferential SESCN. Despite the sample size is small, but this study still includes the largest number of cases till now, and we believed the quality of the data warrant serious consideration of our findings. Besides, we also found the risk factors of the incidence of deep submucosal invasion and angiolymphatic invasion for circumferential SESCN. However, there are still several limitations in the present study. First, this study was conducted at a single institution and a retrospective exploratory design. Additional multicentre and prospective studies with high quality, large sample sizes, and strict operations are required for further verification. Second, the follow-up period of some patients was relatively short and the outcomes need to be further analysed and discussed after extending the follow-up period. Thus, confirmation studies with larger multi-institutional population and adequate follow-up duration are required to confirm the feasibility, safety and suitable criteria of ESD for circumferential SESCN. Nevertheless, the results of this study may provide a useful foundation for future studies.

In conclusion, on the premise of using appropriate measures to prevent postoperative stenosis, ESD could provide better perioperative outcomes in terms of operative time, perioperative complications, and QOL compared with surgery for patients with circumferential SESCN. Meanwhile, the present study demonstrates that there was no significant difference in recurrence rate and mortality between surgery and ESD. It is appropriate to use ESD to treat circumferential SESCN for those elderly patients or patients with poor basic physical conditions who cannot tolerate surgical treatment. Such patients could benefit more from ESD than surgery. The present results might provide endoscopists with useful information for preprocedural decision-making for circumferential SESCN.

## Figures and Tables

**Table 1:** Univariate and multivariate logistic regression analysis for depth of invasion.

Clinical and Histopathologic Characteristics	Depth of Invasion		Univariate Logistic Regression		Multivariate Logistic Regression	
	EP/LPM/MM/SM (<200 μm)	SM (≥200 μm)	OR (95% CI)	*p*	OR (95% CI)	*p*
Age, mean ± SD, years	63.4 ± 7.73	60.9 ± 6.79	0.957 (0.909–1.008)	0.099		
Sex						
Female	38	7	1			
Male	66	29	2.385 (0.954–5.966)	0.063		
Smoking history	40	24	3.200 (1.441–7.105)	0.004		
Drinking history	42	25	3.355 (1.492–7.543)	0.003	3.142 (1.161–8.503)	0.024
Family history of cancer	22	15	2.372 (1.046–5.379)	0.039	3.029 (1.086–8.446)	0.034
Complicated with early laryngeal tumour	6	4	2.552 (0.730–8.926)	0.142		
Lesion location						
Upper	9	2	0.323 (0.064–1.642)	0.173		
Upper-Middle	18	6	0.485 (0.166–1.416)	0.185		
Middle	38	3	0.115 (0.031–0.419)	0.001		
Middle-Lower	32	22	1			
Lower	7	3	0.623 (0.145–2.677)	0.525		
Longitudinal diameter, mm						
≤50	32	8	1.556 (0.639–3.785)	0.330		
>50	72	28				
Macroscopic type						
0-IIa	23	20	4.348 (1.945–9.720)	<0.001		
0-IIb	80	16	1			
0-IIc	1	0	0 (0–∞)	1.000		
WLE						
Hyperkeratosis present	34	16	1.647 (0.759–3.574)	0.207		
Red mucosa	84	34	4.048 (0.897–18.270)	0.069		
Slightly elevated/depressed	24	20	4.167 (1.872–9.274)	<0.001	3.164 (1.201–8.337)	0.020
ME-NBI						
IPCL (JES classification)						
B1	74	7	1		1	
B2/B3	30	29	10.219 (4.040–25.849)	<0.001	10.667 (3.867–29.429)	<0.001
Avascular (AVA) present	30	14	1.570 (0.710–3.469)	0.265		
Degree of differentiation						
Well differentiated	1	1	2.952 (0.177–49.316)	0.451		
Moderately differentiated	62	21	1			
Poorly differentiated	16	12	2.214 (0.903–5.431)	0.082		
Basaloid	3	2	1.968 (0.308–12.598)	0.475		

EP, epithelium, high-grade intraepithelial neoplasia in WHO classification of digestive system tumours; LPM, lamina propria mucosa; MM, muscularis mucosa; SM, submucosa; WLE, white light endoscopy; ME-NBI, Magnifying endoscopy with narrow band imagining; IPCL, intrapapillary capillary loop; OR, odds ratio; CI, confidence interval.

**Table 2:** Univariate and multivariate logistic regression analysis for angiolymphatic invasion.

Clinical and Histopathologic Characteristics	Angiolymphatic Invasion		Univariate Logistic Regression		Multivariate Logistic Regression	
	Absent (−)	Present (+)	OR (95% CI)	*p*	OR (95% CI)	*p*
Age, mean ± SD, years	62.9 ± 7.59	62.1 ± 7.46	0.987 (0.930–1.048)	0.670		
Sex						
Female	41	4	1			
Male	76	19	2.562 (0.817–8.038)	0.107		
Smoking history	47	17	4.220 (1.550–11.487)	0.005	3.385 (1.197–9.571)	0.021
Drinking history	50	17	3.797 (1.396–10.322)	0.009		
Family history of cancer	32	4	0.559 (0.177–1.770)	0.323		
Complicated with early laryngeal tumour	5	5	6.222 (1.636–23.663)	0.007		
Lesion location						
Upper	10	1	0.440 (0.050–3.843)	0.458		
Upper-Middle	19	5	0.811 (0.348–3.847)	0.811		
Middle	36	5	0.611 (0.192–1.950)	0.406		
Middle-Lower	44	10	1			
Lower	8	2	1.100 (0.202–5.990)	0.912		
Longitudinal diameter, mm						
≤50	31	9	0.561 (0.221–1.425)	0.224		
>50	86	14				
Macroscopic type						
0-IIa	31	12	2.991 (1.197–7.473)	0.019		
0-IIb	85	11	1			
0-IIc	1	0	0 (0–∞)	1.000		
WLE						
Hyperkeratosis present	43	7	0.753 (0.287–1.975)	0.564		
Red mucosa	97	21	2.165 (0.470–9.980)	0.322		
Slightly elevated/depressed	32	12	2.898 (1.162–7.225)	0.022		
ME-NBI						
IPCL (JES classification)						
B1	74	7	1		1	
B2/B3	43	16	3.934 (1.499–10.319)	0.005	2.864 (1.035–7.923)	0.043
Avascular (AVA) present	37	7	0.946 (0.359–2.495)	0.911		
Degree of differentiation						
Well differentiated	2	0	0 (0–∞)	0.999		
Moderately differentiated	68	15	1			
Poorly differentiated	21	7	1.511 (0.544–4.199)	0.428		
Basaloid	4	1	1.133 (0.118–10.877)	0.914		
Depth of invasion						
EP/LPM/MM/SM(<200 μm)	96	8	1			
SM(≥200 μm)	21	15	8.571 (3.219–22.824)	<0.001		

EP, epithelium, high-grade intraepithelial neoplasia in WHO classification of digestive system tumours; LPM, lamina propria mucosa; MM, muscularis mucosa; SM, submucosa; WLE, white light endoscopy; ME-NBI, Magnifying endoscopy with narrow band imagining; IPCL, intrapapillary capillary loop; OR, odds ratio; CI, confidence interval.

**Table 3:** Comparison of clinicopathological characteristics between the ESD and surgery groups.

Clinicopathological Characteristics	Treatment		*χ* ^2^	*p*
	ESD	Surgery		
Age, mean ± SD, years	63.1 ± 7.75	61.7 ± 6.79		0.361
Sex			1.669	0.196
Male	71	24		
Female	38	7		
BMI, mean ± SD, kg/m^2^	22.9 ± 3.39	23.2 ± 3.21		0.655
Lesion location			-	0.179
Upper	10	1		
Upper-Middle	22	2		
Middle	32	9		
Middle-Lower	37	17		
Lower	8	2		
Longitudinal diameter, mm				
Median (P_25_, P_75_)	70 (50, 90)	70 (50, 90)		0.520
Macroscopic type			-	0.362
0-IIa	30	13		
0-IIb	78	18		
0-IIc	1	0		
Degree of differentiation			-	0.814
Well differentiated	2	0		
Moderately differentiated	63	20		
Poorly differentiated	19	9		
Basaloid	4	1		
Depth of invasion			7.883	0.005
EP/LPM/MM/SM(<200 μm)	87	17		
SM(≥200 μm)	22	14		
Angiolymphatic invasion present	17	5	-	1.000

EP, epithelium, high-grade intraepithelial neoplasia in WHO classification of digestive system tumours; LPM, lamina propria mucosa; MM, muscularis mucosa; SM, submucosa.

**Table 4:** Clinical outcomes of ESD and surgery.

	Treatment		*p*
	ESD	Surgery	
Total	109	31	
En bloc resection	109 (100.0%)	-	
Complete resection	108 (99.1%)	-	
Operative time			
mean ± SD, minutes	174.5 ± 51.16	255.9 ± 88.18	<0.001
Perioperative complications	6 (5.5%)	6 (19.4%)	0.015
Delayed bleeding	3	0	
Immediate/Delayed Perforation	3	-	
Anastomotic fistula	-	1	
Wound infection	-	5	
Esophageal scar stenosis	84 (77.1%)	-	
Anastomotic stricture	-	4 (12.9%)	
Duration of follow-up (months)			
Median	29.7	39.3	
Range	3.38–78.52	3.75–79.51	
Further treatment			
Radiotherapy	8	-	
Surgery	3	-	
Recurrence	2	3	
Mortality	2	3	

ESD, endoscopic submucosal dissection.

**Table 5:** Functional and symptom scales of the EORTC-QLQ-C30 and the EORTC-QLQ-OES18 (mean ± SD).

	ESD (n = 107)	Surgery (n = 28)	*p*
EORTC-QLQ-C30 Functional scales			
Physical functioning	99.8 ± 1.11	99.5 ± 1.75	0.283
Role functioning	100.0 ± 0.00	100.0 ± 0.00	-
Emotional functioning	99.8 ± 1.13	94.6 ± 10.46	0.014
Cognitive functioning	99.7 ± 2.27	96.4 ± 6.96	0.021
Social functioning	100.00 ± 0.00	100.00 ± 0.00	-
Global health status	85.3 ± 1.92	78.1 ± 9.10	<0.001
EORTC-QLQ-C30 Symptom scales			
Fatigue	0.0 ± 0.00	0.4 ± 2.10	0.326
Nausea and vomiting	0.2 ± 1.61	0.0 ± 0.00	0.611
Pain	0.0 ± 0.00	14.3 ± 18.54	<0.001
Dyspnoea	0.3 ± 3.22	13.1 ± 18.90	0.001
Insomnia	0.0 ± 0.00	20.2 ± 24.58	<0.001
Appetite loss	0.0 ± 0.00	11.9 ± 20.72	0.005
Constipation	0.6 ± 4.55	3.6 ± 13.88	0.277
Diarrhoea	0.3 ± 3.22	17.9 ± 24.82	0.001
Financial difficulties	0.0 ± 0.00	0.0 ± 0.00	-
EORTC-QLQ-OES18 Symptom scales			
Dysphagia	96.3 ± 6.09	95.6 ± 9.72	0.673
Eating difficulties	1.7 ± 4.24	0.9 ± 4.72	0.375
Reflux	3.1 ± 8.30	17.9 ± 22.19	0.002
Esophageal pain	0.4 ± 2.12	6.7 ± 8.73	0.001
Trouble swallowing saliva	0.0 ± 0.00	0.0 ± 0.00	-
Choking when swallowing	8.1 ± 15.08	3.6 ± 10.50	0.071
Dry mouth	0.0 ± 0.00	0.0 ± 0.00	-
Trouble with taste	2.2 ± 8.28	26.2 ± 31.89	<0.001
Trouble with coughing	0.0 ± 0.00	0.0 ± 0.00	-
Speech difficulties	0.3 ± 3.22	0.0 ± 0.00	0.611

EORTC, European Organization for Research and Treatment of Cancer; QLQ-C30, Quality of Life Questionnaire-Core 30; QLQ-OES18, Quality of Life Questionnaire-OES18; SD, standard deviation.

**Table 6:** Comparison of postoperative stenosis expansion times between different interventions groups.

	Repeated EBD	PGA with AEM	PGA with TSI	PGA with AEM and TSI	PGA with AEM and SSWB
Stenosis					
No	0	0	1	20	4
Yes	50 (100.0%)	2 (100.0%)	4 (80.0%)	23 (53.5%)	5 (55.6%)
Balloon dilatation					
mean ± SD	10.8 ± 8.28	14.5 ± 3.54	4.6 ± 4.16	2.9 ± 4.05	3.1 ± 3.52
*p*		0.539	0.104	<0.001	0.008

ESD, endoscopic submucosal dissection; EBD, endoscopic balloon dilation; PGA, polyglycolic acid; AEM, autologous esophageal mucosa; TSI, temporary stent implantation; SSWB, self-control stricture-preventing water balloon.

## Data Availability

The data that support the findings of this study are available from the corresponding author, upon reasonable request.

## Supplementary Materials

The following supporting information can be downloaded at: https://www.mdpi.com/article/10.3390/jcm12020471/s1, Table S1. Clinical and histopathologic characteristics of the patients; Table S2. Comparison of preoperative EUS and postoperative pathology; Table S3. The relationship between preoperative EUS and postoperative pathology.
