# Supplementary material for: The Feasibility and Safety of Endoscopic Submucosal Dissection for Circumferential Superficial Esophageal Squamous Cell Neoplasms
Source: J Clin Med. 2023 Jan 6;12(2):471. doi: 10.3390/jcm12020471 (PMC9860878; doi:10.3390/jcm12020471)
Supplement: Supplementary file 1 [file jcm-12-00471-s001.zip › jcm-2129219-supplementary.pdf]

Supplemental Table S1. Clinical and histopathologic characteristics of the patients

Clinical and histopathologic characteristics	Number of patients (N=140)
Age, mean ±SD (range), years	62.74± 7.55 (45-85)
Sex	
Male	95 (67.9%)
Female	45 (32.1%)
Smoking history	64 (45.7%)
Drinking history	67 (47.9%)
Family history of cancer	36 (25.7%)
Complicated with early laryngeal tumour	10 (7.1%)
Lesion location	
Upper	11 (7.9%)
Upper-Middle	24 (17.1%)
Middle	41 (29.3%)
Middle-Lower	54 (38.6%)
Lower	10 (7.1%)
Longitudinal diameter, mm	
Median（P_{25},P_{75}）	70 (50,90)
Macroscopic type	
0-Ⅱa	43 (30.7%)
0-Ⅱb	96 (68.6%)
0-Ⅱc	1 (0.7%)
White light endoscopy (WLE)	
Hyperkeratosis present	50 (35.7%)
Red mucosa	118 (84.3%)
Slightly elevated/depressed	44 (31.4%)
ME-NBI	
IPCL (JES classification)	
B1	81 (57.9%)
B2	54 (38.6%)
B3	5 (3.6%)
Avascular (AVA) present	44 (31.4%)
Degree of differentiation	
Well differentiated	2 (1.4%)
Moderately differentiated	83 (59.3%)
Poorly differentiated	28 (20.0%)
Basaloid	5 (3.6%)
Depth of invasion	
EP	22 (15.7%)
LPM/MM	76 (54.3%)

SM（<200μm）	6 (4.3%)
SM（≥200μm）	36 (25.7%)
Angiolymphatic invasion	23 (16.4%)
Treatment	
ESD	109 (77.9%)
Surgery	31 (22.1%)

EP, epithelium, high-grade intraepithelial neoplasia in WHO classification of digestive system tumours; LPM, lamina propria mucosa; MM, muscularis mucosa; SM, submucosa; ME-NBI, magnifying endoscopy with narrow band imagining.

Supplemental Table S2. Comparison of preoperative EUS and postoperative pathology

	Preoperative EUS	Postoperative consistency	Accuracy	Insufficient judgements	Excessive judgements
Total	130	77	59.2%		
<SM	61	49	80.3%	12 (19.7%)	
≥SM	69	28	40.6%		41 (59.4%)

SM, submucosa.

Supplement Table S3. The relationship between preoperative EUS and postoperative pathology

Preoperative	Postoperative pathology		Univariate logistic regression	
EUS	EP/LPM/MM	SM	OR (95% CI)	*P*
<SM	49	12	1	
≥SM	41	28	2.789 (1.261-6.165)	0.011

EP, epithelium, high-grade intraepithelial neoplasia in WHO classification of digestive system tumours; LPM, lamina propria mucosa; MM, muscularis mucosa; SM, submucosa.
